# Craniofacial Morphology and Dental Age Assessment in Pediatric Craniosynostosis: A Comparative Cross‐Sectional Study

**DOI:** 10.1002/cre2.70445

**Published:** 2026-09-14

**Authors:** Nutthakarn Ratanasereeprasert, Yanisa Wongbanthit, Narin Intarak, Preeya Suwanwitid, Chutimont Teekavanich, Anand Marya, Guang Hong, Thantrira Porntaveetus

**Affiliations:** ^1^ Department of Orthodontics, Faculty of Dentistry Chulalongkorn University Bangkok Thailand; ^2^ Center of Excellence in Precision Medicine and Digital Health, Department of Physiology, Faculty of Dentistry Chulalongkorn University Bangkok Thailand; ^3^ Clinic of General, Special Care and Geriatric Dentistry, Center for Dental Medicine University of Zurich Zurich Switzerland; ^4^ Orthodontics, Faculty of Dentistry University of Puthisastra Phnom Penh Cambodia; ^5^ Division for Globalization Initiative, Graduate School of Dentistry Tohoku University Sendai Miyagi Japan

**Keywords:** cephalometry, craniosynostosis, dental maturity, healthcare access, maxillary hypoplasia, Thai children

## Abstract

**Objectives:**

To delineate the craniofacial morphometric characteristics of Thai children with syndromic (SC) and non‐syndromic craniosynostosis (NSC) and to assess dental maturation relative to Chronological Age (CA).

**Methods:**

This comparative cross‐sectional study evaluated 19 patients (SC: *n* = 13; NSC: *n* = 6; mean age 9.32 ± 2.67 years). Seventeen skeletal and dental cephalometric variables were analyzed against age‐matched ethnic norms. Dental age (DA) was quantified using the modified Demirjian's four‐tooth method, calibrated via the Thai‐specific Duangto model. One‐sample and Welch's *t*‐tests compared DA with CA and skeletal parameters with normative data.

**Results:**

SC patients demonstrated significant maxillary retrusion and midface hypoplasia, characterized by reduced SNA (73.63 ± 9.52°), ANB (−7.1 ± 10.51°), and maxillary depth (79.36 ± 11.2°) compared to normative data (*p* < 0.05). Additionally, the SC group exhibited a hyperdivergent growth pattern (increased FMA: 32.61 ± 6.87°), a shortened anterior cranial base (41.47 ± 4.56 mm), and compensatory mandibular incisor retroclination (IMPA: 88.38 ± 8.57°) (*p* < 0.05). In contrast, the NSC phenotype was primarily localized to a shortened anterior cranial base (48.17 ± 5.66 mm, *p* < 0.05), with other skeletal parameters remaining within normative ranges. When evaluating dental maturation relative to chronological age, no statistically significant differences were detected in either cohort (mean difference: SC = 0.09 ± 1.50 years, *p* > 0.05; NSC = 1.23 ± 1.61 years, *p* > 0.05).

**Conclusions:**

Children with SC present with severe multi‐planar skeletal discrepancies and secondary dental compensations, whereas NSC‐related dysmorphology is primarily confined to the cranial base. No statistically significant differences were detected between DA and CA in either group. Because dental development does not necessarily reflect the extent of craniofacial skeletal discrepancies, treatment timing must instead rely on comprehensive, patient‐specific structures and skeletal malocclusion.

## 1 Introduction

Craniosynostosis is a congenital craniofacial anomaly characterized by the premature fusion of one or more cranial sutures, resulting in abnormal skull morphology, altered craniofacial growth, and, in some cases, increased intracranial pressure. The condition affects approximately 1 in 2100–2500 live births, with variations across populations (Katouni et al. 2023). Clinically, craniosynostosis is classified as syndromic or non‐syndromic. Syndromic craniosynostosis (SC) is associated with genetic syndromes such as Apert, Crouzon, Pfeiffer, and craniofrontonasal dysplasia, often accompanied by systemic anomalies (Kyprianou and Chatzigianni 2018; Ohazama et al. 2010). In contrast, non‐syndromic craniosynostosis (NSC) typically presents as an isolated defect, most commonly involving the coronal suture, although multiple sutures may also be affected (Lu et al. 2020).

The etiology of craniosynostosis reflects a complex interplay between genetic and environmental factors. Variants in *FGFR1, FGFR2, FGFR3, EFNB1*, and *TWIST1* are well‐recognized drivers of syndromic forms (Intarak et al. 2019; Caengprasath et al. 2021), while intrauterine constraint, maternal smoking, alcohol consumption, and teratogenic exposures have been implicated in non‐syndromic cases. Regardless of cause, premature suture fusion alters craniofacial growth trajectories. SC frequently manifests with midface hypoplasia, shortened cranial base, and skeletal Class III malocclusion, often accompanied by proptosis, airway compromise, and auditory deficits (Reitsma et al. 2013; Faria‐Teixeira et al. 2024; Thaweesapphithak et al. 2022). NSC usually presents with less severe but still clinically relevant craniofacial discrepancies (Kanchanasevee et al. 2020; Intarak et al. 2018).

Cephalometric analysis remains a cornerstone in assessing craniofacial morphology, providing standardized angular and linear parameters to quantify maxillomandibular relationships and growth patterns. Alterations such as reduced SNA and ANB angles, reflecting maxillary retrusion, are well documented in SC (Hariri et al. 2024; Choi et al. 2022). Children with Apert and Crouzon syndromes often present with significant midface deficiency, increased lower facial height, and mandibular rotation compared with unaffected peers (Reitsma et al. 2013). However, most evidence derives from Western populations, and ethnic variation in craniofacial morphology underscores the need for population‐specific data. In Thailand, comprehensive cephalometric studies of craniosynostosis patients are lacking, limiting the ability of craniofacial teams to establish accurate diagnostic references and tailored treatment plans.

Dental development is another critical aspect of patient management. Individuals with craniosynostosis frequently present with anomalies such as agenesis, supernumerary teeth, and crowding, which increase susceptibility to dental caries and periodontal disease (López‐Estudillo et al. 2017; Intarak et al. 2023). In addition, studies have demonstrated delayed dental maturation in SC, particularly in Apert syndrome, compared with both NSC and healthy controls (Reitsma et al. 2013; Chaiboonyarak et al. 2024). These findings highlight the clinical importance of assessing dental age (DA) for growth monitoring and treatment planning. Demirjian's method, based on radiographic evaluation of crown and root development, remains one of the most widely used and reliable approaches for estimating DA (Reitsma et al. 2013). Nevertheless, systematic data on dental development in craniosynostosis patients remain scarce, particularly in Asian populations.

The objectives of this study were to: (1) delineate the craniofacial phenotypes of Thai children with SC and NSC relative to ethnic norms; and (2) evaluate the relationship between dental maturity and chronological age (CA) using the Demirjian method calibrated for the Thai population. By establishing population‐specific morphometric and developmental baselines, this research aims to provide the evidence required to optimize the timing of multidisciplinary interventions and enhance long‐term treatment outcomes.

## 2 Materials and Methods

### 2.1 Study Design and Participant Selection

This retrospective cross‐sectional study was conducted using digital lateral cephalometric and panoramic radiographs archived between 2020 and 2025 at King Chulalongkorn Memorial Hospital and the Faculty of Dentistry, Chulalongkorn University. This study received ethical approval from the Human Research Ethics Committee of the Faculty of Dentistry, Chulalongkorn University (HREC‐DCU 2026‐007) and in accordance with the 1964 Helsinki declaration and its later amendments. Prior to data collection, formal written informed consent was obtained from the parents or legal guardians of all participating minors. The reporting of this study conforms to the Strengthening the Reporting of Observational Studies in Epidemiology (STROBE) guidelines (Supporting file 1).

The study population comprised 19 Thai children (11 females, 8 males) aged 6–15 years (mean age 9.32 ± 2.67 years) diagnosed with craniosynostosis. Participants were categorized into two groups based on clinical examination, genetic testing, and imaging:

1. Syndromic craniosynostosis (SC, *n* = 13): Including Apert, Crouzon, Pfeiffer, and Craniofacial frontonasal dysplasia.
2. Non‐syndromic craniosynostosis (NSC, *n* = 6): Including uni‐coronal and multi‐suture synostosis.

All the patients have no history of orthodontic treatment.

### 2.2 Cephalometric Morphometry

Standardized lateral cephalometric radiographs were obtained with the subjects in the Frankfort horizontal plane, teeth in maximum intercuspation, and lips in repose. Seventeen skeletal and dental variables were digitized and analyzed using specialized software.

**Figure 1:**
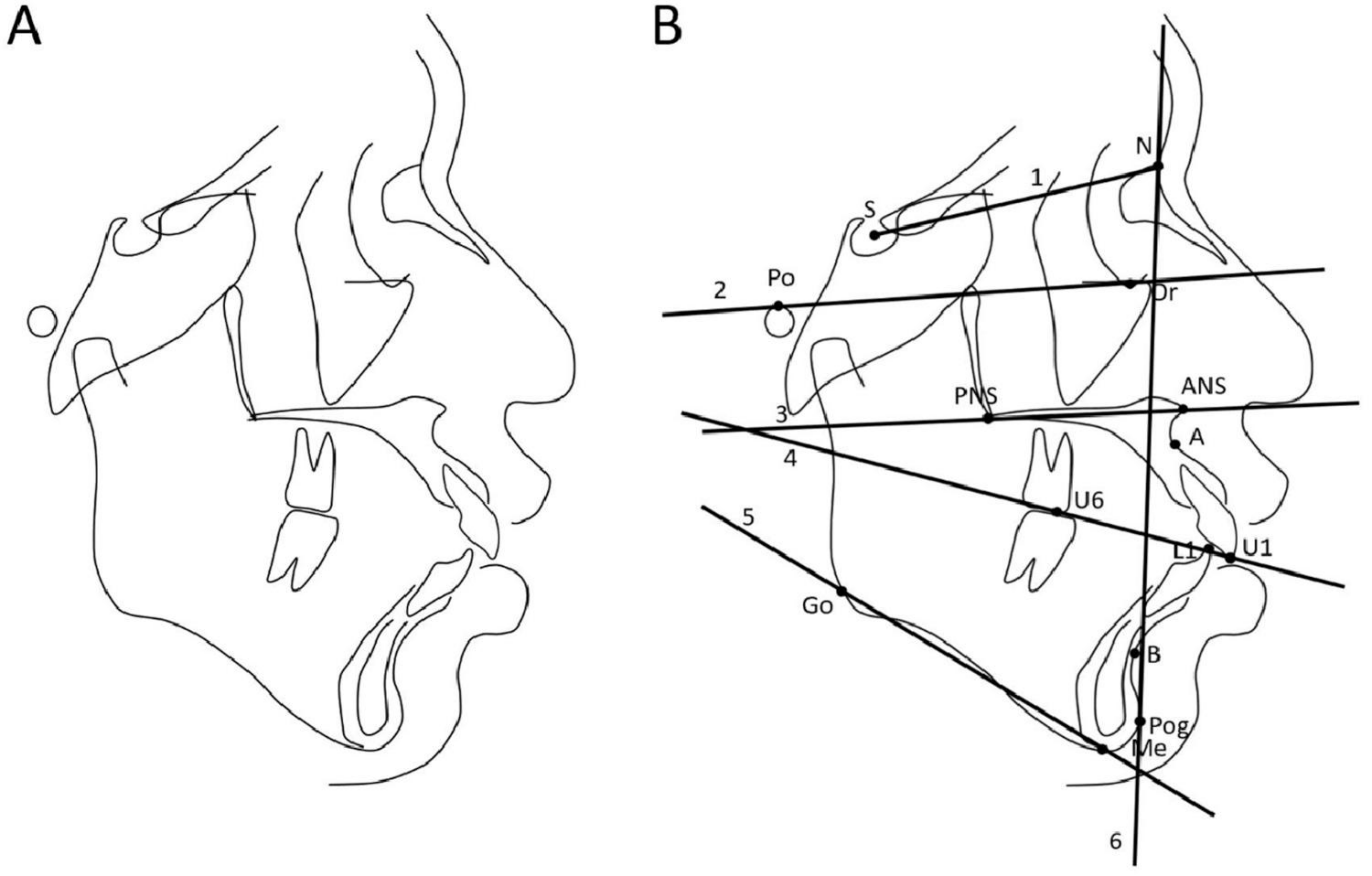
Cephalometric landmarks and reference planes. Abbreviations, full names, and definitions. (A) cephalometric landmarks; N (Nasion); S (Sella); Or (Orbitale); Po (Porion); A (Point A); ANS (Anterior nasal spine); PNS (Posterior nasal spine); B (Point B); U1 (incisal edge of the maxillary central incisor); U6 (mesiobuccal cusp tip of maxillary first molar); L1 (incisal edge of the mandibular central incisor); GO (Gonion); ME (Menton); POG (Pogonion). (B) Reference planes; SN plane (Sella‐Nasion plane); FH plane (Frankfort horizontal plane); PP plane (Palatal plane); Occlusal plane; Mandibular plane; N‐Pog plane.

Cephalometric landmarks (e.g., Sella, Nasion, Point A, Point B) and reference planes (e.g., SN plane, Palatal plane, Mandibular plane) were identified to quantify multi‐planar craniofacial morphology (Figure 1). To ensure diagnostic accuracy, two calibrated orthodontists (NR, PS) performed the measurements. Intra‐ and inter‐examiner reliability were assessed via a second round of measurements following a 2‐week washout period.

### 2.3 Dental Maturity and Age Estimation

Dental development was evaluated from panoramic radiographs using the modified Demirjian four‐tooth method (mandibular right canine, first premolar, second premolar, and second molar) (Demirjian and Goldstein 1976). Developmental stages (A–H) were recorded and converted into maturity scores.

To determine the Dental Age (DA) relative to the Thai population, scores were calibrated using the Duangto model (Duangto et al. 2018), a validated age‐prediction equation for Thai children. This allowed for a direct comparison between DA and the subjects’ Chronological Age (CA). Two calibrated orthodontists (NR, CT) independently evaluated tooth developmental stages. To ensure diagnostic consistency, all radiographs were re‐scored after a 2‐week washout period. Intra‐ and inter‐examiner reliability were subsequently quantified using Cohen's Kappa statistics.

### 2.4 Statistical Analysis

In cephalometric assessment, intra‐examiner reliability and inter‐examiner reliability were determined using the intraclass correlation coefficient. Then, statistical analyses were performed using the Shapiro–Wilk test to assess the normality of the data on the cephalometric measurement and the dental maturity score of SC and NSC patients (R Foundation for Statistical Computing, Vienna, Austria). If the data were normally distributed, for the cephalometric values, the one‐sample *t*‐test was applied to compare differences between the SC and norm group, and NSC and norm group. Welch's *t*‐test was used to compare SC and NSC.

As for the dental maturity stages, intra‐examiner reliability and inter‐examiner reliability were assessed using Cohen's Kappa statistics. After dental maturity stages were converted into dental maturity scores, these were further converted to DA using the new equation for age estimation in Thai children by Duangto et al (2018). Statistical analyses were performed using the Shapiro–Wilk test to assess the normality of the data on the DA of SC and NSC patients. The Wilcoxon signed rank test was used to determine the significant difference between the DA of SC patients and their real CAs, and NSC patients and their real CAs. A *p*‐value of less than 0.05 was considered statistically significant.

## 3 Results

### 3.1 Participant Characteristics and Surgical History

The final study cohort comprised 19 participants, comprising 11 females (57.9%) and 8 males (42.1%) with a mean age of 9.32 ± 2.67 years at the time of radiographic assessment. The sample included 13 patients with SC, specifically Apert, Crouzon, Pfeiffer, and Craniofacial frontonasal dysplasia, and 6 patients with NSC, including unicoronal and multisuture involvement.

Surgical intervention was documented in 18 of the 19 patients. Primary operations were performed at a mean age of 1.4 years (6 months–5 years). The primary procedures included fronto‐orbital advancement (FOA, *n* = 9), cranial vault remodeling (CVR, *n* = 5), and FOA combined with ancillary procedures (*n* = 4). Eight patients (42.1%) required secondary operations at a mean age of 4.1 years (1.1–10 years). None of the participants had a history of orthodontic treatment, allowing for the assessment of natural dental compensations.

Detailed surgical histories were documented, including primary CVR or FOA are shown in Table 1.

**Table 1:** Clinical data of patients with syndromic and non‐syndromic craniosynostosis and corresponding surgical procedures.

Patient ID	SC or NSC	Age	Gender	Clinical diagnosis	1st surgery	2nd surgery
P01	SC	7	Male	Frontonasal syndrome with left coronal craniosynostosis	FOA + SOT at 1 yr 6 mo	MOT at 3 yr 6 mo
P02	SC	7	Male	Multiple sutures craniosynostosis	FOA + posterior CVR at 3 yr	—
P03	SC	7	Female	Apert syndrome	FOA at 1 yr	—
P04	SC	8	Female	Right coronal synostosis	Anterior CVR at 5 yr	—
P05	SC	8	Male	Pfeiffer syndrome	Posterior CVR at 8 mo	FOA at 1 yr 2 mo
P06	SC	9	Male	Crouzon syndrome	CVR at 1 yr	—
P07	SC	9	Female	Crouzon syndrome	FOA at 6 mo	Posterior CVR at 2 yr 4 mo
P08	SC	10	Female	Craniofrontonasal dysplasia with coronal craniosynostosis	FOA + MOT at 1 yr	redo MOT at 8 yr
P09	SC	10	Male	Left coronal craniosynostosis	—	—
P10	SC	11	Male	Apert syndrome	FOA at 2 yr	—
P11	SC	12	Male	Apert syndrome	FOA at 1 yr	—
P12	SC	13	Male	Crouzon syndrome	FOA at 10 mo	—
P13	SC	15	Female	Apert syndrome	FOA at 1 yr	re‐FOA at 10 yr
P14	NSC	6	Female	Multiple sutures craniosynostosis	FOA at 1 yr 2 mo	anterior and middle CVR at 5 yr
P15	NSC	8	Female	Multiple suture craniosynostosis	Posterior CVR at 7 mo	FOA at 1 yr 9 mo
P16	NSC	8	Female	Multiple suture craniosynostosis	FOA at 1 yr	—
P17	NSC	9	Female	Right coronal synostosis	FOA at 10 mo	frontal bone repositioning at 1 yr 1 mo
P18	NSC	10	Female	Microcephaly with bilateral coronal craniosynostosis	Posterior CVR at 3 yr	—
P19	NSC	14	Female	Multiple suture craniosynostosis	FOA + posterior CVR at 1 yr	—

Abbreviations: CVR, cranial vault remodeling; FOA, fronto‐orbital advancement; MOT, medial orbital translocation; mo, month; NSC, non‐syndromic craniosynostosis; SC, syndromic craniosynostosis; SOT, superior orbital translocation; yr, year.

### 3.2 Reliability Analysis

Statistical evaluation of examiner reliability indicated excellent reproducibility across all parameters. For cephalometric digitization, intra‐rater Intraclass Correlation Coefficients (ICC) were > 0.90 for both examiners, while inter‐rater ICC exceeded 0.80, reflecting strong consistency in landmark identification. Dental maturity staging demonstrated high concordance, with both intra‐ and inter‐rater Cohen's Kappa values surpassing 0.80. These values are categorized as almost perfect agreement, confirming that the radiographic assessments were highly reproducible and free from significant systemic bias between sessions or examiners.

### 3.3 Craniofacial Morphometry

**Figure 2:**
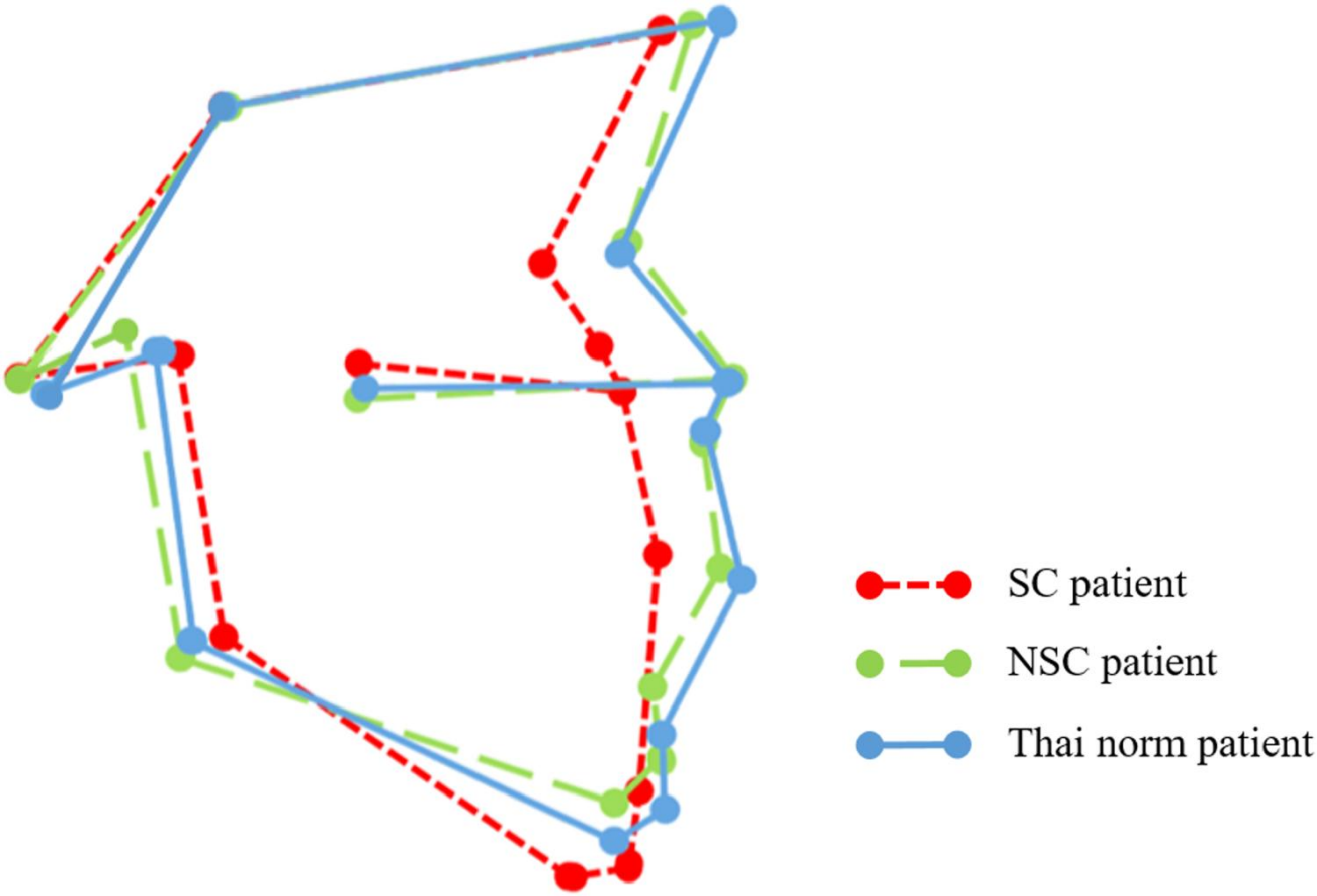
The Comparative illustration of the morphological craniofacial bone anomalies of patients with SC (red lines), NSC (green lines), and Thai norm patient (blue lines).

Cephalometric analysis identified distinct phenotypic signatures for the SC and NSC cohorts compared to ethnic normative data. Comparative morphometric data and phenotypic illustrations are presented in Table 2 and Figure 2, respectively.

**Table 2:** Means, standard deviations, and *p*‐values for cephalometric measurements: one‐sample *t*‐tests comparing SC with Thai norms and NSC with Thai norms, and Welch's *t*‐test comparing SC and NSC groups. A statistical difference was determined at *p* ≤ 0.05.

Measurements	SC		Thai norm			NSC		Thai norm			SC		NSC		
	Mean	SD	Mean	SD	*p*‐value	Mean	SD	Mean	SD	*p‐*value	Mean	SD	Mean	SD	*p*‐value
Skeletal cephalometric measurements															
SNA (°)	73.63	9.52	83	4	0.004*	84.19	6.89	83	4	0.691	73.63	9.52	84.19	6.89	0.017*
SNB (°)	80.73	4.51	79	3	0.192	79.65	3.28	79	3	0.65	80.73	4.51	79.65	3.28	0.564
ANB (°)	−7.10	10.51	4	2	0.003*	4.54	4.98	4	2	0.801	−7.10	10.51	4.54	4.98	0.005*
Wits (mm)	−11.76	10.57	−3	2	0.011*	−0.79	2.59	−3	2	0.091	−11.76	10.57	−0.79	2.59	0.003*
SN‐GoGn (°)	37.02	8.38	34	6	0.218	30.71	7.20	34	6	0.314	37.02	8.38	30.71	7.20	0.119
FMA (°)	32.61	6.87	25	4	0.002*	23.98	5.37	25	4	0.661	32.61	6.87	23.98	5.37	0.011*
Anterior cranial base length (S‐N) (mm)	41.47	4.56	55	2.7	< 0.001*	48.17	5.66	55	2.7	0.032*	41.47	4.56	48.17	5.66	0.033*
Maxillary depth (FH‐NA) (°)	79.36	11.20	90.3	3.3	0.004*	93.31	8.73	90.3	3.3	0.436	79.36	11.20	93.31	8.73	0.012*
Facial depth (FH‐NPog) (°)	86.12	3.45	85	3.2	0.906	89.52	3.50	85	3.2	0.057	86.12	3.45	89.52	3.50	0.077
Convexity of point A (A‐NPog) (mm)	−3.84	6.42	4.5	2.3	0.001*	3.04	4.98	4.5	2.3	0.505	−3.84	6.42	3.04	4.98	0.025*
Cranial deflection (°)	29.17	3.71	28.7	1.6	0.654	29.23	2.78	28.7	1.6	0.661	29.17	3.71	29.23	2.78	0.971
Dental cephalometric measurements															
IMPA (°)	88.38	8.57	99	4	0.001*	96.75	9.71	99	4	0.595	88.38	8.57	96.75	9.71	0.104
L1‐NB (°)	28.61	9.55	32	6	0.224	29.50	11.34	32	6	0.612	28.61	9.55	29.50	11.34	0.871
L1‐NB (mm)	6.60	2.94	6	2	0.479	5.65	3.98	6	2	0.836	6.60	2.94	5.65	3.98	0.616
U1‐NA (°)	35.96	15.21	28	4	0.084	27.83	15.48	28	4	0.98	35.96	15.21	27.83	15.48	0.311
U1‐NA (mm)	9.10	5.65	5	3	0.023*	6.33	4.54	5	3	0.504	9.10	5.65	6.33	4.54	0.277
U1‐L1 (°)	122.68	16.51	118	8	0.327	117.42	11.73	118	8	0.908	122.68	16.51	117.42	11.73	0.441

*Note:* References for Lateral Cephalometric Thai Norm: 1. Sorathesn K. Craniofacial norm for Thai in combined orthodontic surgical procedure. J Dent Assoc Thai. 1988;38(5):190–201. 2. Suchato W, Chaiwat J. [Assessment of normal craniofacial and dental relationships in Thai people]. Mahidol Dent J. 1984;34(5):233–243. 3. Sutthiprapaporn P, Manosudprasit A, Pisek A, Manosudprasit M, Pisek P, Phaoseree N. Establishing esthetic lateral cephalometric values for Thai adults after orthodontic treatment. Khon Kaen Dent J. 2020;23(2):31–41. Statistical significance was defined as a *p*‐value < 0.05 or *p < 0.05.

### 3.4 SC Versus Thai Norm

Cephalometric analysis identified distinct phenotypic signatures for the SC and NSC cohorts compared to ethnic normative data. Comparative morphometric data and phenotypic illustrations are presented in Table 2 and Figure 2, respectively.

The SC group exhibited generalized multi‐planar craniofacial dysmorphology, with statistically significant deviations from normative values in 53% (9/17) of the assessed parameters. A profound midface deficiency was observed, characterized by a significant reduction in SNA, maxillary depth, and convexity of point A. This resulted in a skeletal Class III relationship confirmed by markedly negative ANB and Wits appraisal values (*p* < 0.05). Vertically, these patients exhibited a hyperdivergent growth pattern, with a significantly increased FMA, while the anterior cranial base length was significantly shorter than the Thai norm, suggesting an early constraint on the template for midfacial development. To mitigate the skeletal discrepancy, significant dental compensation was observed through the proclination of maxillary incisors (increased U1‐NA distance) and compensatory retroclination of mandibular incisors (decreased IMPA).

### 3.5 NSC Versus Thai Norm

In contrast to the SC group, the NSC group maintained a craniofacial profile largely conformed to Thai normative values. The primary morphometric anomaly in this group was a significantly shortened anterior cranial base. However, other sagittal and vertical parameters, including maxillary position and incisor inclinations, showed no significant deviation from the norm. These findings suggest that the phenotypic expression of NSC in this population is primarily localized to the cranial vault and base with relative sparing of the midfacial and mandibular complexes.

### 3.6 SC Versus NSC

Direct inter‐group comparison between the two craniosynostosis cohorts identified seven key skeletal variables that differentiated the phenotypes. The SC group demonstrated significantly more severe maxillary hypoplasia and a more pronounced concave facial profile (convexity of point A) compared to the NSC group, as evidenced by significantly lower SNA, ANB, and Wits appraisal values.

Furthermore, both the hyperdivergent skeletal tendency (increased FMA) and the shortening of the anterior cranial base were significantly more pronounced in the SC cohort. Despite these marked differences in skeletal base severity, no statistically significant differences were observed in dental inclinations or positions between the SC and NSC groups, which suggests a common threshold for physiological dental compensation across both phenotypes.

### 3.7 DA Assessment

DA was determined using a Thai‐specific age prediction model (Duangto et al. 2018). The Wilcoxon signed‐rank test indicated that no statistically significant difference was detected between DA and CA in either the SC group (mean difference = 0.09 ± 1.50 years, *p* > 0.05) or the NSC group (mean difference = 1.23 ± 1.61 years, *p* > 0.05).

## 4 Discussion

### 4.1 Cephalometric Radiographic Findings and Skeletal Morphology

This study indicates that Thai children with SC exhibit pronounced midface hypoplasia characterized by significant maxillary retrognathia and a concave facial profile. This morphological signature aligns with the established pathophysiology of syndromes such as Apert and Crouzon, where premature synostosis extends beyond the cranial vault to involve the circum‐maxillary suture systems (Kreiborg and Aduss 1987; Alam et al. 2023). A pivotal observation in our cohort was the significant attenuation of the anterior cranial base in both the SC and NSC groups. As the cranial base serves as the primary structural scaffold for midfacial development, its shortening likely serves as a fundamental driver of the observed maxillary retrusion (Enlow and Azuma 1975).

Figure 2 provides a qualitative schematic illustration of the primary multi‐planar skeletal discrepancies across the cohorts. It is intended for visual conceptualization of phenotypic patterns and does not represent a mathematically averaged cephalometric superimposition. For precise dimensional and angular variations, readers should refer to the quantified data presented in Table 2.

This morphological pattern conforms to the principle of growth restriction, in which bone development is inhibited in the plane perpendicular to the involved suture. Since SC frequently involves the coronal suture complex, the resulting restriction in the anteroposterior dimension of the skull is a predictable consequence of the primary pathology (Lun et al. 2022). Furthermore, the hyperdivergent skeletal pattern identified in the syndromic group, evidenced by an increased FMA, suggests a significant clockwise rotation of the mandible (Wu et al. 2020). This vertical discrepancy may represent functional compensation for restricted maxillary volume or may arise from altered muscular vectors and soft‐tissue constraints inherent to these complex syndromes (Nitayavardhana and Srichomthong 2020). The resilience of the mandibular growth trajectory compared to the restricted maxillary growth further exacerbates the skeletal Class III malocclusion.

### 4.2 Odontogenesis and DA Assessment

Our data reveal no significant discrepancy between dental maturity and CA in either the SC or NSC cohorts (Katouni et al. 2023). Despite the systemic severity of these syndromes, the frequency of invasive surgical interventions, and the presence of localized dental anomalies, including crowding, ectopic eruption, and agenesis (Wongbanthit et al. 2024; Porntaveetus et al. 2017), dental maturation scores in this sample remained closely aligned with chronological references.

The precision of these developmental findings was bolstered by a pragmatic adaptation of the Demirjian four‐tooth system (Demirjian and Goldstein 1976), which focused specifically on the mandibular right canine, premolars, and second molar. This methodological modification was essential to accommodate the high prevalence of hypodontia and tooth agenesis, particularly involving the lower lateral incisors and left‐quadrant units, frequently observed in craniosynostosis populations. By integrating this right‐sided assessment with the Duangto age‐prediction model (Duangto et al. 2018), we ensured a robust analysis that remained inclusive of patients with severe dental anomalies while utilizing an ethnic‐specific developmental benchmark calibrated for the Thai population.

These findings stand in notable contrast to existing international literature. For instance, studies in Dutch cohorts have reported significant delays in tooth formation among patients with Apert and Crouzon syndromes (Reitsma et al. 2014), while others have observed accelerated dental maturation in single‐suture, non‐syndromic cases (Leinonen et al. 2021). The alignment between DA and CA in our sample may be related to the application of the Duangto model, a developmental benchmark specifically calibrated for the Thai population (Duangto et al. 2018). By utilizing an ethnic‐specific reference, we likely mitigated the systematic errors associated with the standard Demirjian method, which was derived from a French‐Canadian sample and may not accurately reflect Southeast Asian maturation rates. Furthermore, the high reliability of this approach, demonstrated by ICC and Kappa values exceeding 0.80, confirms that DA can be accurately and consistently assessed even in the presence of complex craniofacial and dental anomalies.

### 4.3 Clinical and Surgical Relevance

Interpreting these craniofacial phenotypes requires accounting for early surgical intervention as a potential confounder of post‐natal growth. In this cohort, 18 of 19 patients underwent major primary reconstructions, including FOA or CVR, at a mean age of 1.4 years. While these procedures are critical for intracranial decompression and morphological restoration, the extensive nature of the surgery and subsequent re‐operations may introduce iatrogenic constraints on long‐term development. Specifically, periosteal disruption and contractile scar tissue formation have been hypothesized to impede forward maxillary displacement, potentially exacerbating midface hypoplasia (Meazzini et al. 2011).

However, our comparative analysis suggests that despite undergoing similar surgical protocols, the NSC cohort maintained maxillary positions consistent with Thai normative data, whereas the SC group exhibited profound skeletal Class III patterns. This phenotypic divergence highlights a strong association between syndromic conditions and midfacial retrusion, suggesting that intrinsic biological factors, in addition to surgical history, may contribute to these distinct skeletal profiles. These findings underscore the dominance of the underlying syndromic phenotype over external clinical interventions in shaping the final skeletal Class III malocclusion.

From a clinical perspective, these findings emphasize the critical role of individualized, multidisciplinary treatment planning for Thai children with craniosynostosis. The profound midface hypoplasia identified in syndromic cases necessitates early surgical strategies, such as LeFort III or Monobloc distraction osteogenesis, to address functional imperatives including airway patency, orbital protection, and sagittal skeletal harmony (Zheng et al. 2025; Rattan et al. 2024).

Notwithstanding the profound structural constraints of the skeletal base, the dentoalveolar complex exhibited a robust adaptive capacity (Kawasaki et al. 2014). The SC cohort demonstrated significant maxillary incisor proclination coupled with mandibular incisor retroclination, representing a definitive physiological adaptation to maintain functional occlusion within a skeletal Class III framework. These findings align with previous literature and case reports documenting similar compensatory mechanisms in various craniosynostosis populations (Carpentier et al. 2014; Shin et al. 2020). While these natural adaptations may provide transient functional benefits during mixed dentition, they must be meticulously managed; intentional orthodontic decompensation will be required in late adolescence to achieve stable skeletal and dental relationships if definitive orthognathic surgery is pursued (Shin et al. 2020).

Crucially, the preservation of dental maturation despite severe skeletal disharmony reinforces the clinical reality that dental development is not a stable indicator for orthodontic timing. Instead, clinicians must navigate the dissociation between skeletal and dental maturity by adopting a case‐specific approach that integrates the patient's unique dental phenotype, the severity of malocclusion, and the specific surgical trajectory to optimize both functional and esthetic outcomes in this complex population (Flores‐Mir et al. 2004).

### 4.4 Study Limitations and Future Directions

Several limitations warrant consideration when interpreting these results. First, the inherent rarity of SC necessitated a relatively small sample size, which may constrain the generalizability of our morphometric findings to the broader population. Second, the cohort's broad age range (6–15 years) encompasses significant developmental transitions, including the mixed dentition stage and the pubertal growth spurt; this chronological diversity introduces inevitable variability in skeletal and dental positioning. Furthermore, our reliance on two‐dimensional (2D) cephalometry limits the assessment of three‐dimensional (3D) volumetric asymmetries and functional airway alterations that are often central to these phenotypes. Future research utilizing longitudinal 3D imaging (CBCT) and larger multi‐center cohorts is required to further elucidate the complex interplay between surgical intervention and intrinsic growth potential.

Future investigations utilizing Cone Beam Computed Tomography are warranted to provide a more comprehensive characterization of 3D sutural morphology and functional spatial relationships. Such high‐resolution imaging would offer a more nuanced understanding of the interplay between skeletal architecture and physiologic development in this patient population.

## 5 Conclusions

This study demonstrates that Thai children with syndromic SC exhibit severe multi‐planar skeletal dysmorphology, primarily characterized by profound midface hypoplasia and hyperdivergent growth, whereas the NSC phenotype is largely restricted to anterior cranial base shortening. Despite these structural discrepancies, no statistically significant differences were detected between DA and CA in either group. Because dental maturation does not reliably correspond to the severity of craniofacial skeletal deformities, clinical decision‐making and the timing of orthodontic and surgical interventions require an integrated assessment of patient‐specific skeletal maturity, functional requirements, and long‐term growth potential.

## Author Contributions

Conceptualization, Nutthakarn Ratanasereeprasert and Thantrira Porntaveetus. Investigation, Nutthakarn Ratanasereeprasert, Yanisa Wongbanthit, Preeya Suwanwitid, and Chutimont Teekavanich. Resources, Nutthakarn Ratanasereeprasert, Yanisa Wongbanthit, Preeya Suwanwitid, and Chutimont Teekavanich. Data Curation, Nutthakarn Ratanasereeprasert, Narin Intarak, Anand Marya, and Guang Hong. Writing – Original Draft, Nutthakarn Ratanasereeprasert and Thantrira Porntaveetus. Writing – Review and Editing, Nutthakarn Ratanasereeprasert, Narin Intarak, and Thantrira Porntaveetus. Supervision, Thantrira Porntaveetus. All authors read and gave their final approval and agreed to be accountable for all aspects of the work.

## Ethics Statement

This study received ethical approval from the Human Research Ethics Committee of the Faculty of Dentistry, Chulalongkorn University (HREC‐DCU 2026−007) and in accordance with the 1964 Helsinki declaration and its later amendments.

## Consent

The authors have nothing to report.

## Conflicts of Interest

The authors declare no conflicts of interest.

## Supporting information


**Supporting File:** STROBE Checklist — Checklist of items that should be included in reports of cross‐sectional studies.

## Data Availability

Original data generated and analyzed during this study are included in this published article or in the supporting information.
