# Supplementary material for: Craniofacial Morphology and Dental Age Assessment in Pediatric Craniosynostosis: A Comparative Cross‐Sectional Study
Source: Clin Exp Dent Res. 2026 Sep 14;12(5):e70445. doi: 10.1002/cre2.70445 (PMC13573083; doi:10.1002/cre2.70445)
Supplement: Supplementary file 1 [file CRE2-12-e70445-s001.docx]

**Supplementary file 1**

STROBE Statement—checklist of items that should be included in reports of cross-sectional studies

|  | Item No. | Recommendation | Page  No. | Relevant text from manuscript |
| --- | --- | --- | --- | --- |
| **Title and abstract** | 1 | (*a*) Indicate the study’s design with a commonly used term in the title or the abstract | 1 | Craniofacial Morphology and Dental Age Assessment in Pediatric Craniosynostosis: A Comparative Cross-Sectional Study |
|  |  | (*b*) Provide in the abstract an informative and balanced summary of what was done and what was found | 2-3 | Complete abstract section, detailing objectives, methods, quantitative cephalometric and dental age results, and conclusions. |
| Introduction | | | |  |
| Background/rationale | 2 | Explain the scientific background and rationale for the investigation being reported | 4-5 | Paragraphs 1–4: Outlines the genetic/environmental etiology of craniofacial synostosis, the roll of SC vs. NSC phenotypes, the gap in Southeast Asian data, and the role of dental maturation analysis. |
| Objectives | 3 | State specific objectives, including any prespecified hypotheses | 5 | (1) delineate the craniofacial phenotypes of Thai children with syndromic and non-syndromic craniosynostosis relative to ethnic norms; (2) assess the concordance between dental maturity and chronological age using the Demirjian method. |
| Methods | | | |  |
| Study design | 4 | Present key elements of study design early in the paper | 6 | This retrospective cross-sectional study was conducted using digital lateral cephalometric and panoramic radiographs archived between 2020 and 2025. |
| Setting | 5 | Describe the setting, locations, and relevant dates, including periods of recruitment, exposure, follow-up, and data collection | 6 | King Chulalongkorn Memorial Hospital and the Faculty of Dentistry, Chulalongkorn University |
| Participants | 6 | *Cross-sectional study*—Give the eligibility criteria, and the sources and methods of selection of participants | 6 | The study population comprised 19 Thai children. Participants were categorized into two groups (SC and NSC) based on clinical examination, genetic testing, and imaging. All the patients have no history of orthodontic treatment. |
| Variables | 7 | Clearly define all outcomes, exposures, predictors, potential confounders, and effect modifiers. Give diagnostic criteria, if applicable | 7 | Outcomes: 17 skeletal and dental cephalometric variables, and Dental Age |
| Data sources/ measurement | 8* | For each variable of interest, give sources of data and details of methods of assessment (measurement). Describe comparability of assessment methods if there is more than one group | 7 | Standardized lateral cephalometric radiographs analysed for landmarks and Dental maturation evaluated using the modified Demirjian four-tooth method calibrated to the Thai-specific Duangto model. |
| Bias | 9 | Describe any efforts to address potential sources of bias | 7-8 | Intra- and inter-examiner reliability were assessed via a second round of measurements following a two-week washout period. |
| Study size | 10 | Explain how the study size was arrived at | 6 | Sample size determined by convenience sampling of rare clinical archives. |

Continued on next page

| Quantitative variables | 11 | Explain how quantitative variables were handled in the analyses. If applicable, describe which groupings were chosen and why | 7-8 | Continuous cephalometric degrees/millimeters and dental age scores were evaluated for normality via the Shapiro-Wilk test. Subjects were explicitly grouped into SC and NSC phenotypes. |
| --- | --- | --- | --- | --- |
| Statistical methods | 12 | (*a*) Describe all statistical methods, including those used to control for confounding | 7-8 | Shapiro-Wilk test, one-sample t-test (vs. norms), Welch’s t-test (inter-group SC vs. NSC comparison). |
|  |  | (*b*) Describe any methods used to examine subgroups and interactions | 7-8 | Shapiro-Wilk test, one-sample t-test (vs. norms), Welch’s t-test (inter-group SC vs. NSC comparison). |
|  |  | (*c*) Explain how missing data were addressed | 7-8 | Complete case analysis; no missing data encountered. |
|  |  | (*d*) *Cross-sectional study*—If applicable, describe analytical methods taking account of sampling strategy | 7-8 | Wilcoxon signed-rank test utilized to evaluate paired deviations between dental age and chronological age |
|  |  | (*e*) Describe any sensitivity analyses | - | Sensitivity analyses not performed. |
| Results | | | | |
| Participants | 13* | (a) Report numbers of individuals at each stage of study—eg numbers potentially eligible, examined for eligibility, confirmed eligible, included in the study, completing follow-up, and analysed | 6 | study cohort comprised 19 participants, comprising 13 patients with SC and 6 patients with NSC. |
|  |  | (b) Give reasons for non-participation at each stage | - | Retrospective archive study; exclusions/flowcharts were omitted due to total cohort use. |
|  |  | (c) Consider use of a flow diagram | - | Retrospective archive study; exclusions/flowcharts were omitted due to total cohort use. |
| Descriptive data | 14* | (a) Give characteristics of study participants (eg demographic, clinical, social) and information on exposures and potential confounders | 6-7 Table 1 | Clinical demographics, clinical diagnoses, and extensive details on primary/secondary surgeries (FOA, CVR, mean age 1.4 years) are provided in the text and comprehensive Table1 |
|  |  | (b) Indicate number of participants with missing data for each variable of interest | - | No missing data across parameters. |
| Outcome data | 15* | Report numbers of outcome events or summary measures | Table 2 | Summary measures for all 17 cephalometric outcomes are fully presented across SC, NSC, and Thai norm cohorts in **Table 2**. Dental age mean differences are reported in the **Dental Age Assessment** text. |
| Main results | 16 | (*a*) Give unadjusted estimates and, if applicable, confounder-adjusted estimates and their precision (eg, 95% confidence interval). Make clear which confounders were adjusted for and why they were included | Table 2 | **Table 2** lists exact Means, SDs, and *p*-values for all inter-cohort and normative comparison permutations (p < 0.05 marked with *). |
|  |  | (*b*) Report category boundaries when continuous variables were categorized | Table 2 | Categorization defined strictly by clinical/genetic syndrome diagnostic classification lines (SC vs. NSC). |

| Other analyses | 17 | Report other analyses done—eg analyses of subgroups and interactions, and sensitivity analyses | 9 | **Reliability Analysis:** Quantifies intra-examiner and inter-examiner reproducibility with Intraclass Correlation Coefficients (ICC > 0.90 and > 0.80) and Cohen's Kappa (> 0.80). |
| --- | --- | --- | --- | --- |
| Discussion | | | | |
| Key results | 18 | Summarise key results with reference to study objectives | 12-15 | Paragraph 1 & 4: Recaps severe multi-planar midface hypoplasia in SC, localized cranial base defects in NSC, and the distinct cross-sectional alignment between dental age and chronological age. |
| Limitations | 19 | Discuss limitations of the study, taking into account sources of potential bias or imprecision. Discuss both direction and magnitude of any potential bias | 16 | Paragraph 8: Thoroughly breaks down study limitations including small sample size (n = 19), developmental growth variations across the wide age group (6–15 years), and 2D radiographic constraints. |
| Interpretation | 20 | Give a cautious overall interpretation of results considering objectives, limitations, multiplicity of analyses, results from similar studies, and other relevant evidence | 12-16 | Paragraphs 2–7: Synthesizes spatial skeletal features against chronological dental concordance, evaluates surgical confounding, and clarifies that dental development cannot dictate orthodontic timing. |
| Generalisability | 21 | Discuss the generalisability (external validity) of the study results | 16 | Paragraph 8: Explicitly notes that the small sample size may limit the generalizability of these morphometric findings across the wider spectrum of rare craniosynostosis phenotypes. |
| Other information | |  | | |
| Funding | 22 | Give the source of funding and the role of the funders for the present study and, if applicable, for the original study on which the present article is based | 18 | Funding is included in the Declarations. |

*Give information separately for cases and controls in case-control studies and, if applicable, for exposed and unexposed groups in cohort and cross-sectional studies.

**Note:** An Explanation and Elaboration article discusses each checklist item and gives methodological background and published examples of transparent reporting. The STROBE checklist is best used in conjunction with this article (freely available on the Web sites of PLoS Medicine at http://www.plosmedicine.org/, Annals of Internal Medicine at http://www.annals.org/, and Epidemiology at http://www.epidem.com/). Information on the STROBE Initiative is available at www.strobe-statement.org.
